# Interactions of the Skin Pathogen *Haemophilus ducreyi* With the Human Host

**DOI:** 10.3389/fimmu.2020.615402

**Published:** 2021-02-03

**Authors:** Julie A. Brothwell, Brad Griesenauer, Li Chen, Stanley M. Spinola

**Affiliations:** ^1^ Department of Microbiology and Immunology, Indiana University School of Medicine, Indianapolis, IN, United States; ^2^ Department of Medicine, Indiana University School of Medicine, Indianapolis, IN, United States; ^3^ Department of Pathology and Laboratory Medicine, Indiana University School of Medicine, Indianapolis, IN, United States

**Keywords:** *Haemophilus ducreyi*, interactome, metabolome, skin, immune response

## Abstract

The obligate human pathogen *Haemophilus ducreyi* causes both cutaneous ulcers in children and sexually transmitted genital ulcers (chancroid) in adults. Pathogenesis is dependent on avoiding phagocytosis and exploiting the suppurative granuloma-like niche, which contains a myriad of innate immune cells and memory T cells. Despite this immune infiltrate, long-lived immune protection does not develop against repeated *H. ducreyi* infections—even with the same strain. Most of what we know about infectious skin diseases comes from naturally occurring infections and/or animal models; however, for *H. ducreyi*, this information comes from an experimental model of infection in human volunteers that was developed nearly three decades ago. The model mirrors the progression of natural disease and serves as a valuable tool to determine the composition of the immune cell infiltrate early in disease and to identify host and bacterial factors that are required for the establishment of infection and disease progression. Most recently, holistic investigation of the experimentally infected skin microenvironment using multiple “omics” techniques has revealed that non-canonical bacterial virulence factors, such as genes involved in central metabolism, may be relevant to disease progression. Thus, the immune system not only defends the host against *H. ducreyi*, but also dictates the nutrient availability for the invading bacteria, which must adapt their gene expression to exploit the inflammatory metabolic niche. These findings have broadened our view of the host-pathogen interaction network from considering only classical, effector-based virulence paradigms to include adaptations to the metabolic environment. How both host and bacterial factors interact to determine infection outcome is a current focus in the field. Here, we review what we have learned from experimental *H. ducreyi* infection about host-pathogen interactions, make comparisons to what is known for other skin pathogens, and discuss how novel technologies will deepen our understanding of this infection.

## Introduction

As a primary barrier to infection, the skin possesses multiple defense mechanisms including antimicrobial peptides, immune cells, and the commensal microbiome. These factors, among others, contribute to inter-person variability in response to infection. Studies investigating natural infection outcomes have been very useful for determining pathology later in infection (1), but are limited in their ability to inform what happens early in infection, since patients generally do not seek care at these times. Differences in host factors as well as different strains of a given pathogen, which may have varying degrees of virulence, further confound understanding how the immune system responds to infection.

The skin immune response differs among pathogens despite the general conservation of cell types that either reside in and patrol the skin or that are recruited to combat the infectious agent. Much of what we know about skin immunology has been inferred from murine infection models or by comparing datasets from convenience human skin samples obtained from plastic surgery (2) and from biopsy of diseased skin (3, 4). Both strategies have elucidated important players in the skin immune response, but have notable caveats. While animal models allow for better controlled studies, the skin of mice differs from human skin in terms of thickness, time to healing, microbiome, and immune response, which can all be attributed to differences in both genetics and environments. Thus, interpretations of animal studies are limited to pathogenesis and immune mechanisms shared by both the animal model and humans. Extrapolation of differential gene expression between diseased and either healthy or reference datasets from different anatomical locales, such as convenience human tissue samples, can have similar limitations.

To circumvent these limitations and further our understanding of how human skin responds to infection in a controlled fashion, we have used experimental skin infection of human volunteers with the extracellular, Gram-negative bacterium *Haemophilus ducreyi* for nearly thirty years (5, 6). *H. ducreyi* is the causative agent of two diseases: the sexually transmitted genital ulcer (GU) disease chancroid and cutaneous ulcers (CU) in children (7). Chancroid is a sexually transmitted genital ulcer disease and facilitates the transmission of HIV-1 (8). Due to syndromic management, defined as the provision of treatment without diagnostic testing, the epidemiology of chancroid is not well understood, but chancroid remains endemic in developing countries *via* reservoirs of infected commercial sex workers (7). Recently, in tropical or equatorial regions with high prevalence of CU caused by *Treponema pallidum* subsp. *pertenue* (yaws), *H. ducreyi* was found to be the etiological agent in ~40-60% of lesions (9–11). For both yaws and *H. ducreyi*, the ulcers are primarily found on the lower leg and affect approximately 4-10% of children in endemic regions. While the prevalence of *H. ducreyi*-associated CU initially decreased following mass drug administration of azithromycin, the disease was not eliminated due to environmental reservoirs (12, 13). Thus, there is a continuing need to understand *H. ducreyi* pathogenesis and the host response to this infection.

There are two classes of *H. ducreyi* isolates; class I and II strains can be differentiated based on several variable extracellular or secreted proteins (DsrA, NcaA, DltA, LspA1, LspA2, and others), and lipooligosaccharide (LOS) structure (14), but are otherwise highly conserved, clinically indistinguishable, and found in significant proportions of both CU and GU (12, 15–17). Although the majority of CU are typically caused by a single genotype, coinfections with both classes are common in CU (12); such studies have not been done in GU. Most *in vitro* work and experimental human infection utilizes *H. ducreyi* 35000HP, a human-passaged class I GU strain. By whole genome sequencing, ~70% of CU strains are nearly identical to 35000HP, indicating that our model is highly relevant to both GU and CU (15, 16).

Although both class I and II strains cause CU and GU, whether individual isolates can cause both diseases within a population is unknown due to a lack of surveillance of GU in CU endemic areas and *vice versa*. In addition, the number of HD isolates that have been sequenced is quite limited, and syndromic management of GU precludes our understanding of how prevalent each class is in the population. Whether there are differences in infectivity or the ability each class to cause disease in different anatomical locales in natural infection is unknown. To date, 35000HP is the only strain used in experimental human infection. Since 35000HP is a GU isolate and able to infect nongenital skin following the same disease kinetics and pathology seen in GU, it appears that its pathogenic potential is similar in genital and nongenital skin.

Here, we highlight and discuss the recent advances made in understanding *H. ducreyi* pathogenesis *via* mutant versus parent comparison trials, the immune response to *H. ducreyi* infection, and how *H. ducreyi* may be exploiting the immune system to promote its pathogenesis.

## Overview of the Human Challenge Model

**Figure 1:**
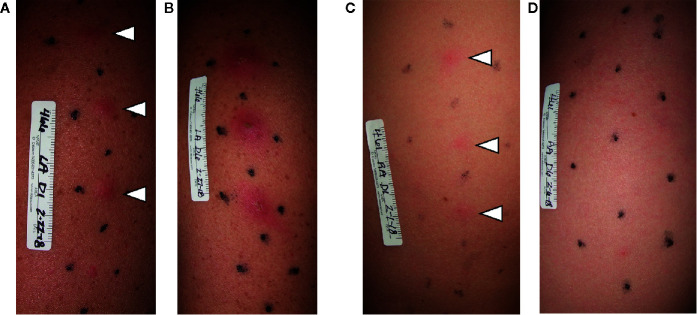
Photographs of clinical outcomes in the *H*. *ducreyi* human challenge model. Volunteers were infected in three sites and mock infected in the fourth, bottom-most site. On day 1 (24 hpi), both volunteers had papules at all three infected sites (**A**, **C**; white arrowheads). On day 6 of infection, the volunteer in **(A)** had developed pustules at all infected sites **(B)**. In contrast, by day 6, the volunteer in **(C)** had resolved infection at all three infected sites **(D)**.

The *H. ducreyi* experimental infection model is arguably one of the best studied human infection model systems. It is also a powerful model for investigating bacterial pathogenesis due to FDA approval for not only experimental infection of wild type and mutant bacterial strains but also the ability to perform punch biopsies of infected sites. In this model, as few as one bacterium introduced into wounded skin on the upper arm is sufficient to cause infection (18). This suggests that experimental human infection mimics natural infection—especially considering that infection of animal models requires 3–6 log-fold higher inocula. Although most individuals experimentally infected with wild type *H. ducreyi* form papules within 24 hours of infection, only ~70% of individuals progress to the pustular stage; the other ~30% spontaneously resolve the infection within 2 to 5 days (5) (**Figure 1**). The clinical endpoints are resolution of infection or development of a painful pustule; volunteers with pustules (N=231) are infected for 8.1 ± 2.4 (mean ± SD) days. As subject safety considerations preclude progression of experimental infection to the ulcerative stage, experimental infection mirrors natural disease only until the pustular stage. Pustule formation is dependent on host effects and gender, as men are twice as likely to form pustules as women (5). Re-infection of individuals from past *H. ducreyi* challenge studies demonstrated that individuals who initially formed pustules tend to form pustules and individuals who resolved infection the first time tend to resolve infection a second time at different statistically significant rates (19). This was not attributable to serum bactericidal or opsonizing antibodies. Similarly, in patients with natural chancroid, there is no apparent protection to re-infection. Although the underlying molecular mechanisms leading to pustule formation or resolution are unknown, varying degrees of immune tolerance—particularly with regard to dendritic cells (DCs)—are hypothesized to play a role.

To date, 34 mutants have been tested in the human model to define which virulence factors are required for infection (**Table 1**). The methods for these double-blinded, multi-stage, dose ranging studies have been reviewed previously (5). Mutants are categorized as virulent (form pustules at doses similar to the parent), partially attenuated (form pustules at doses 2- or 3-fold that of the parent, but not at doses equivalent to the parent) or as attenuated (unable to form pustules even at doses 10-fold that of the parent). Of the 34 mutants, 10 were attenuated, 9 were partially attenuated, and 15 were virulent. The trials have shown the importance of evasion of phagocytosis, resistance to complement mediated killing and antimicrobial peptides, the ability to form aggregates, quorum sensing, adherence to collagen and fibrin, nutrient uptake, and adaptation to starvation as pathogenic mechanisms for disease progression. The interaction of some of these virulence determinants with the host immune response will be discussed in detail below.

**Table 1:** *H. ducreyi* mutants tested in the human challenge model.

Gene	Definition	Function	Results	Source of Mutant(Reference)
*hhdB*	secretion/activation of hemolysin	lyses fibroblasts	Virulent	Munson (20)
*losB*	D-glycero-D-manno-heptosyltransferase	extends LOS beyond KDO-triheptose-glucose	Virulent	Munson (21)
*lst*	lipooligosaccharide sialytransferase	adds sialic acid to major glycoform of LOS	Virulent	Munson (21)
*ftpA*	fine tangled pilus major subunit	unknown	Virulent	Spinola (22)
*hgbA*	hemoglobin binding outer membrane protein	heme/iron acquisition and transport from hemoglobin	Attenuated	Elkins (23)
*momp*	major outer membrane protein	OmpA homologue; minor role in fibronectin binding	Virulent	Spinola & Hansen (24)
*pal*	peptidoglycan associated lipoprotein	lipoprotein; OM stability	Attenuated	Spinola (25)
*dsrA*	ducreyi serum resistance A	OMP; major role in serum resistance	Attenuated	Elkins (26)
*cdtC*	cytolethal distending toxin	toxic for T cells, epithelial cells, fibroblasts	Virulent	Hansen (27)
*cdtC/hhdB*	double mutant, cytolethal distending toxin, hemolysin	toxic for T cells, epithelial cells, fibroblasts; lyses fibroblasts	Virulent	Hansen & Munson (27)
*glu*	glucosyltransferase gene	adds glucose to KDO-triheptose LOS core	Virulent	Campagnari & Munson (28)
*sodC*	superoxide dismutase C	detoxifies ROS for bacteria in phagolysosomes	Virulent	Kawula (29)
*tadA*	tight adherence protein A	type 1V secretion; secretion of Flp proteins	Attenuated	Hansen (30)
*lspA1/lspA2*	double mutant, large supernatant proteins	secreted proteins; prevent Fc-γRmediated phagocytosis	Attenuated	Hansen (31)
*ompP2A/ompP2B*	double mutant, porin proteins	encode known classical trimeric porins	Virulent	Campagnari (32)
*dltA*	ducreyi lectin A	OMP; partial serum resistance	Partially Attenuated	Elkins (33)
*ncaA*	necessary for collagen adherence	OMP; confers collagen binding	Attenuated	Kawula (34)
*wecA*	first enzyme in the ECA biosynthetic pathway	initiates synthesis of putative ECA glycoconjugate	Partially Attenuated	Munson (35)
*tdX/tdhA*	double mutant; ton B dependent receptors	heme uptake	Virulent	Elkins (36)
*fgbA*	putative fibrinogen binding adhesion	OMP that confers binding to fibrinogen	Partially Attenuated	Bauer & Janowicz (37)
*luxS*	homolog of *V. harveyi luxS*	Autoinducer; quorum sensing	Partially Attenuated	Hansen (38)
*sapA*	Susceptibility to antimicrobial peptides	Confers partial resistance to LL-37	Partially Attenuated	Bauer (39)
*cpxA*	Sensor kinase of CpxRA 2-component system	Uncontrolled activation of CpxR	Attenuated	Munson & Spinola (40)
*flp-1,-2,-3*	Fimbria-like proteins 1,2,3	Microcolony formation	Attenuated	Munson (41)
*cpxR*	Response Regulator of CpxRA system	Senses Extracytoplasmic Stress	Virulent	Hansen (42)
*sapBC*	Susceptibility to antimicrobial peptides	Confers resistance to LL-37	Attenuated	Bauer (43)
*neuA*	CMP sialic acid synthetase	Makes required substrate for all sialyltransferases	Virulent	Munson (44)
*csrA*	Carbon storage regulator	Post transcriptional regulator; affects mRNA stability and translation	Partially Attenuated	Spinola (45)
*ompP4*	OMP with homology to *H. influenzae ompP4*	putative lipoprotein expressed *in vivo*	Virulent	Janowicz (46)
*hfq*	Facilitates binding of sRNAs with mRNAs	Post transcriptional regulator	Attenuated	Spinola (47)
*relA spoT* double mutant	Encode enzymes for synthesis/hydrolysis of (p)ppGpp	Transcriptional regulator; mediates the stringent response to starvation	Partially Attenuated	Spinola (48)
35000HP∆PEA (*HD0371*, *HD0852*, *HD1598* triple mutant)	Encode 3 putative PEA transferases	Alters LOS charge; confers resistance to α and β defensins	Virulent	Bauer (49)
*dksA* mutant	RNA polymerase (RNAP)-binding transcription factor	Co-regulator of RNAP with (p)ppGpp; has independent transcriptional effects	Partially Attenuated	Spinola (50)
35000HP*hgbA_II_*	class I *hgbA* allele replaced by a class II allele in 35000HP	Heme/iron acquisition; possible surrogate for class II strains for vaccine studies	Partially Attenuated	Leduc (51)

To our knowledge, the only other active human challenge model using a skin pathogen is the Bacille Calmette-Guérin (BCG) model, which was developed about twelve years ago to assess BCG vaccine efficiency against BCG infection (52). Later the BCG vaccine challenge model was used to evaluate if specific *Mycobacterium tuberculosis* (53) and *M. leprae* (54) proteins were cross-reactive. This model injects BCG into the forearm skin and has also been instrumental in defining the immune infiltrate and cytokines released into the microenvironment (52).

## The Role of the Host During *H. ducreyi* Infection

### Histopathology of the Cutaneous Ulcer

**Figure 2:**
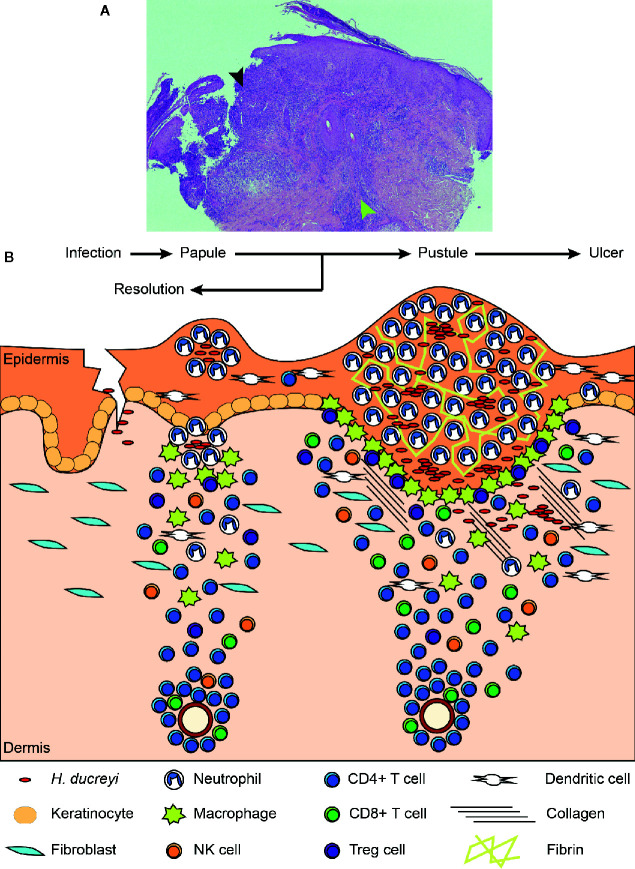
Histology of an experimental lesion and the evolution of disease progression. **(A)** H & E staining of an endpoint pustule. The black arrowhead indicates the site of the pustule, which has eroded through the epidermis. The green arrowhead indicates the mononuclear cell infiltrate in the dermis. **(B)** Schematic of disease progression from time of infection to papule and pustule formation. Details of this process appear in the text. Adapted from Atlas of Sexually Transmitted Diseases and AIDS, 4^th^ edition (56).

Our understanding of the role of the host during disease has been aided by histological examination of both experimental and natural infections (1, 55). A schematic of the progression of disease from the papular to pustular stage is found in **Figure 2**. Infection is dependent on entry through wounds in the skin; intact skin is resistant to infection. *H. ducreyi* first encounters keratinocytes, fibroblasts, tissue resident and memory T cells, and tissue resident DCs in healthy skin but remains extracellular throughout infection (1, 18, 55). Collagen and fibrin are deposited in the wounds, and innate immune cells are recruited to the site of infection (55, 57). Neutrophils initially surround and attempt to phagocytose the bacteria, forming a microabscess in the epidermis and dermis (55, 57). Macrophages concurrently migrate into the tissue and form a “collar” around the base of the abscess (1, 57). Immature DCs, natural killer (NK) cells, T cells, and a few B cells are simultaneously recruited and localize primarily below the macrophage collar (58–61). If the phagocytic response fails to clear the organism, the papule evolves into a pustule that resembles a suppurative granuloma and eventually erodes into a painful ulcer (1).

Immunocytochemistry of papules and pustules revealed that the bacteria are mainly confined to the upper levels of the pustule, where they are surrounded by neutrophils (1, 55, 57). In experimental infection, *H. ducreyi* is localized to the epidermis and upper dermis, which contrasts to the localization of other skin pathogens that form granulomas in the deep dermis. For instance, *Mycobacterium leprae* is an intracellular skin pathogen that lives in macrophages. Similar to *H. ducreyi*, the immune response appears to involve walling off the infection, so the infected macrophages are surrounded by T cells whose subtype determines whether a pro- or anti-inflammatory environment is created (62, 63). Another mycobacterium, *M. ulcerans* remains extracellular due to secretion of mycolactone and, ideally, is prevented from spreading further into the tissue by macrophages like *H. ducreyi*, but ulcers can progress to the deep dermis if the infection cannot be controlled (64).

When considering other genital skin infections, chancroidal ulcers can be macroscopically similar to syphilitic chancres caused by *T. pallidum*. However, in syphilis, bacterial replication is typically in the deep dermis and involves the vasculature (65). Like chancroid, neutrophils are first recruited, but macrophages and the T cells required for infection control are not recruited until late in the second week of infection with *T. pallidum* (65).

**Figure 3:**
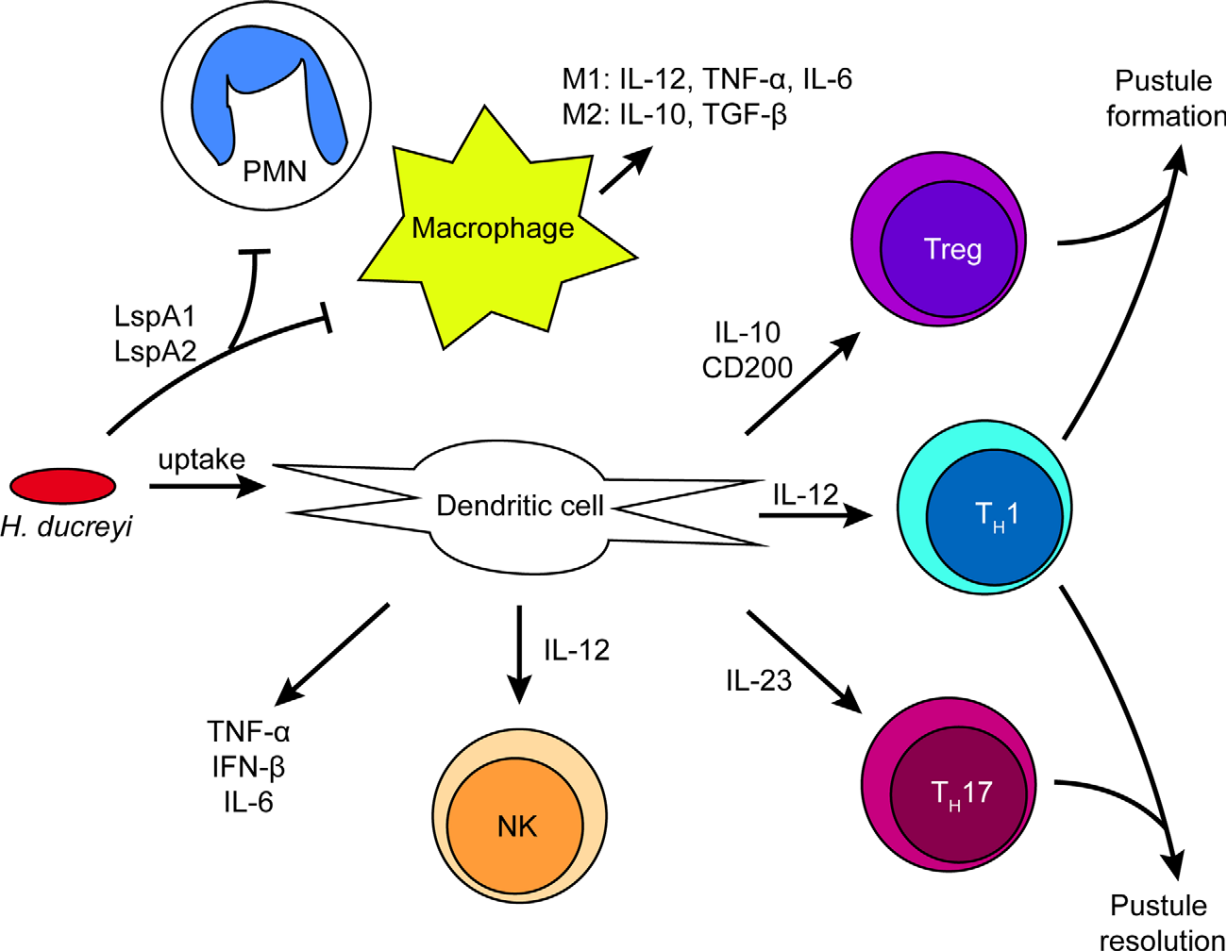
Summary of immune cell activation by *H. ducreyi* in a pustule. Schematic of immune cell interactions and the cytokine environment in experimental infection based on transcriptomics and *in vitro* data. *H. ducreyi* secretes Large supernatant protein (Lsp)A1 and LspA2 to inhibit phagocytosis by neutrophils and macrophages. However, *H. ducreyi* is directly taken up by dendritic cells; ingested bacteria and/or soluble antigens from lysed bacteria such as lipooligosaccharide (LOS) result in dendritic cell activation. Dendritic cells subsequently secrete the indicated cytokines that lead to activation of NK cells and T cell subsets. The ratio of different T cell subsets in the lesion may be correlated with disease outcome. Further details appear in the text.

How immune cells are recruited to the site of infection and how *H. ducreyi* responds to the immune system is discussed below and summarized in **Figure 3**.

### Antigen Presentation and Cytokine Secretion by Dendritic Cells

Myeloid DCs are among the most important antigen presenting cells recruited to sites of *H. ducreyi* infection (58). DCs have both surface and intracellular pattern recognition receptors (PRRs) that recognize foreign molecules or molecules from damaged cells. Ligand binding to PRRs activates DCs, leading to phagocytosis and the transcription of pro- and anti-inflammatory immune response genes that influence the subsequent T cell response. While large supernatant protein (Lsp) A1 and LspA2 have been shown to inhibit phagocytosis of *H. ducreyi* by neutrophils and macrophages (66), their expression does not impact DC uptake of *H. ducreyi* (58). Whether this is because DCs are not as sensitive to phagocytic inhibition by LspA1 and LspA2 or whether DCs use a different mechanism to phagocytose *H. ducreyi* compared to neutrophils and macrophages remains to be determined. DCs can also be activated by *H. ducreyi* LOS in a non-contact-dependent manner (67), indicating that phagocytosis of *H. ducreyi* by DCs is sufficient but not necessary to promote DC activitation. *H. ducreyi* is able to partially inhibit DC activation and maturation through an unknown mechanism; however, DCs are still able to secrete high levels of IL-6 and TNF-α in response to live *H. ducreyi* (58). Secretion of high levels of IL-6 along with partially activated or immature DCs are hypothesized to be mechanisms by which *H. ducreyi* avoids a full adaptive immune response and survives immune onslaught.

DCs present antigen to activate T cells and subsequently secrete cytokines that polarize T cells; however, *H. ducreyi* also partially inhibits DC activation and maturation (58). Despite this, *H. ducreyi-*specific T cell lines have been isolated from pustules of volunteers who have not been previously exposed to *H. ducreyi*, which suggests that DCs are able to present antigen to naïve T cells that differentiate into memory cells (68). Differences in pathogen detection and cytokine production that could influence T cell responses have been examined in experimental *H. ducreyi* infection. Single-nucleotide polymorphisms in Toll-like receptor 9 and interleukin-10 alleles have been identified and are predictive of pustule formation or resolution in experimental infection (69). Microarray analysis of infected and mock infected skin obtained from pustule formers and resolvers who were re-infected for 48 hours suggested that the two groups had both core and distinct tissue transcriptional responses to infection (19). Compared to resolvers, pustule formers had a hyperinflammatory, dysregulated response in the tissue. Monocyte-derived myeloid DCs obtained from resolvers and pustule formers who were re-infected with *H. ducreyi* also shared a core transcriptional response that should promote Th1 responses. However, resolver DC transcriptomes also suggested the promotion of a Th17 response and the upregulation of IL-23, whereas pustule former DCs promoted a Treg response and the upregulation of IL-10 and CD200. LOS binding to TLR4 on DCs leads to production of TNF-α and IFN-β, which induces indolamine 2,3-dioxygenase (IDO) expression, and IDO induction in DCs may explain the increased number of Tregs found in pustules (67). IDO expression in DCs can promote conversion of naïve T cells to Tregs, prevent Treg conversion to effector T cells, inhibit T cell activation/proliferation, and promote T cell death through tryptophan depletion and production of pro-apoptotic metabolites (70–72), which would result in a decrease in the number of effector T cells and an increase in Tregs. These data suggest that *H. ducreyi* infection simultaneously increases both pro-inflammatory and anti-inflammatory cytokines and that the balance between the pro-inflammatory and anti-inflammatory signals in different hosts may dictate whether a pustule forms or resolves.

Along with T cells, DCs also activate NK cells (60). *H. ducreyi-*infected DCs activate NK cells *in vitro* through production of IL-12 and subsequently lead to NK cell secretion of IFN-γ. IFN-γ secretion, primarily originating from NK cells early in infection, could increase immune-mediated phagocytosis of *H. ducreyi*.

### T Cell Response in Infection

In experimental lesions, CD4+ T cells are primarily recruited and localize beneath the macrophage collar; the average CD4:CD8 ratio is 3:1 (61). T cells comprise 50% of leukocytes in papules at two days post infection. Approximately 70% of T cells collected from papules are CD45RO^+^ memory T cells, which are likely a mix of skin-resident memory T cells and circulating central memory T cells that responded to chemokines released at the infected site. Whether the T cells in the papule can respond to *H. ducreyi* has not been tested. We previously observed that DCs in pustule formers and resolvers upregulate different transcripts; these transcripts suggest that the DCs in resolvers promote a type 1 and type 17 response while the DCs in pustule formers promote a type 1 and regulatory response (19). Therefore, we hypothesize that the T cells in the skin of resolvers and pustule formers may similarly differ. In the skin, CD49a (integrin α_1_) expression has been used to differentiate IFN-γ- versus IL-17-expressing CD8^+^ cells, which are CD49a^+^ and CD49a^-^, respectively (73). Although the differences between CD49a^+^ and CD49a^-^ CD4 T cells have yet to be determined, CD4 T cells in diseased skin can also express CD49a^+^ (74). If the T cells identified in papules do respond to *H. ducreyi* either directly or through an unknown cross-reactive memory T cell response, then pustule formers may have more CD49a^+^ T cells and resolvers may have more CD49a^-^ T cells. Alternatively, if the T cells are not responding to *H. ducreyi*, then the T cells that were observed in papules are likely representative of the basal level of T cells in the skin and of non-specific memory T cells that may be following chemotactic gradients.

A hallmark of the adaptive immune system is the recall response. T cell lines have been derived from pustules that were biopsied 6-14 days after experimental infection (68). Treatment of the T cell lines with *H. ducreyi* antigen in the presence of autologous peripheral blood mononuclear cells (PBMCs), but not irradiated allogeneic PBMCs, led to proliferation and cytokine production from T cells, indicating that the response is MHC restricted. The T cell lines produced minimal responses to related members of the *Pasteurellaceae* family, demonstrating that the T cell lines are *H. ducreyi* antigen-specific. Interestingly, this antigen-specific response does not seem to confer protection against re-challenge, as 88% of pustule formers form pustules when re-challenged (75, 76). The reason for the lack of immunity is unclear, but it may in part be due to the lack of antibody responses during experimental infection. Antibody responses are made late in the ulcerative stage of natural infection, but these antibodies are not bactericidal (77).

### Avoiding Phagocytosis by Macrophages and Neutrophils

*H. ducreyi* remains extracellular and prevents its uptake by both neutrophils and macrophages primarily by the secretion of two proteins: LspA1 and LspA2 (66). LspA1 and LspA2 are the largest proteins encoded by the *H. ducreyi* 35000HP genome, have proportionately higher numbers of SNPs compared to most *H. ducreyi* genes (16), and are secreted by the LspB transporter. Secreted LspA1 and LspA2 are taken up by neutrophils and macrophages by an unknown mechanism, bind to and are phosphorylated by the human C-terminal tyrosine kinase Csk, and increase Csk activity *via* a positive feedback loop (78). This increased Csk activity prevents human Src kinases from inducing the actin rearrangements required for FcγR-dependent phagocytosis. Phagocytes with inhibited FcγR-dependent phagocytosis are unable to engulf opsonized bacteria. Ducreyi serum resistance protein A (DsrA) is an outer membrane protein that inhibits IgM from binding to the bacterial surface thereby preventing complement deposition and serum killing (79, 80); theoretically, DsrA could also inhibit complement-mediated phagocytosis. Both DsrA and fibrinogen binding protein A (FgbA) are adhesins that bind fibrinogen, similar to the M proteins of *Streptococcus pyogenes* (37, 81). Although the role of FgbA and DsrA in resistance to phagocytosis has not been established, binding to fibrinogen in other bacteria is thought to help prevent phagocytosis through adhesion to host cells, steric hindrance, and shielding pathogen associated molecular patterns (PAMPs) that would be recognized by phagocytes (82, 83). Deletion mutants of *lspA1* and *lspA2*, *dsrA*, or *fgbA* are attenuated for pustule formation in humans (26, 31, 37). Thus, despite appearing somewhat redundant, they are all required to establish productive infection and, given their posited *in vitro* functions, to avoid phagocytosis.

With so many mechanisms to prevent phagocytosis, how does the immune response manage to help kill the bacteria and resolve infection? Characterization of macrophages from biopsies of *H. ducreyi* infected pustules and from *in vitro H. ducreyi* -infected monocyte-derived macrophages has shown that infection produces a mixed M1/M2 macrophage population (84). The M1 phenotype is characterized by being microbicidal, producing high levels of IL-12, TNF-α, IL-6, and reactive nitrogen and oxygen intermediates; the M2 phenotype is associated with tissue repair and reducing inflammation, producing IL-10 and TGF-β (85). M2 macrophages are also better at phagocytosis than M1 macrophages, especially M2c macrophages, which are polarized by IL-10 and are efficient at phagocytosing *H. ducreyi via* class A scavenger receptors (84). The proportion of M1 versus M2 macrophages may play an important role in resolving *H. ducreyi* infection, but is yet to be studied in the context of pustule formation and resolution. Given that resolvers who are re-infected upregulate IL-10 transcripts in tissue compared to pustule formers (19), we hypothesize that resolvers may have more M2c macrophages than pustule formers and are therefore better able to phagocytose and clear *H. ducreyi* infection *via* class A scavenger receptor uptake.

The ratio of type 1 and type 2 immune cells has been instrumental in predicting disease progression for bacterial pathogens. *M. leprae* infection is dynamic. Type 1 and type 17 cells dominate in tuberculoid lesions and type 2 and regulatory cells dominate in lepromatous lesions; oscillation between these two states during natural disease correlates with changes of immune cell phenotypes (62). Similarly, prevention of *Staphylococcus aureus* and *Streptococcus pyogenes* skin infections requires a predominantly type 1 immune response rather than a type 2 immune response (86–89). Interestingly, the immune cell typing that controls infection of *M. leprae* (90), *S. aureus* (86), or *S. pyogenes* (89) favors skewing towards a predominately type 1 response, whereas the immune cell typing that controls infection of *H. ducreyi* may favor a mixed type 1 and type 17 response (19). Important distinctions between these pathogens may account for this difference. For instance, M2 macrophages express an *M. leprae* entry receptor, CD163 (91), which would explain why leprosy lesions worsen with increased numbers of M2 macrophages. The skewing of macrophage typing towards an M2 response leads to their release of cytokines that can also push the T cells toward a type 2 response.

### Defensin and Cathelicidin Resistance

In addition to avoiding phagocytosis, *H. ducreyi* also resists antimicrobial peptides that are secreted by phagocytes and keratinocytes. *H. ducreyi* encodes two transporters that confer resistance to some human defensins and the cathelicidin LL-37. A proton motive force-dependent transmembrane efflux pump, Mtr, confers resistance to LL-37 and β-defensins—especially HBD-3. A *mtrC* deletion mutant had decreased expression of *lspB* and *dsrA*, indicating that multiple virulence factors are impacted by the absence of MtrC (92). Another transporter system, Sap, also ameliorates LL-37 activity (93). SapB and SapC form a heterodimeric transporter that transports cargo bound by the periplasmic chaperone SapA. A *sapA* deletion mutant is 25-50% more sensitive to killing by LL-37 *in vitro* and has an approximately 50% reduction in pustule formation in the human infection model (39). However, deletion of *sapBC* results in a mutant that is fully attenuated for pustule formation in the human infection model, suggesting that SapBC may be secreting additional SapA-like protein(s) that serve redundant functions (43).

The positive charge of the *H. ducreyi* outer membrane was also hypothesized to repulse antimicrobial peptide binding (49). The outer membrane of most Gram-negative bacteria contains lipopolysaccharide (LPS); however, *H. ducreyi* has a similar, less decorated version—LOS (94). Class I strains have a sialylated nonasaccharide (95), while Class II stains have a non-sialylated pentasaccharide (96). Deletion of either the sialyltransferase *lst* or the N-acylneuraminate cytidylyltransferase *neuA* eliminates LOS sialylation in the Class I strain *H. ducreyi* 35000HP (44). Both mutants are fully virulent and form pustules in human volunteers. This contrasts to the pathogenic *Neisseria* spp. in which sialylation of LOS is a major virulence determinant (97). *H. ducreyi* LOS and core oligosaccharide each contain a positively charged phosphoethanolamine (PEA) group (96, 98), which helps to neutralize the otherwise negatively charged LOS and was hypothesized to repel positively charged LL-37. However, deletion of *lptA*, which attaches PEA to the LOS, did not affect pustule formation (49). Overall, cathelicidin resistance is primarily mediated by the Sap and Mtr transporters rather than membrane charge or sialylation (43, 92).

### Interaction of the Host With *H. ducreyi*

While cytokines and redox species certainly shape the lesion environment, what the microenvironment contains on a molecular level is a current focus of investigation. The molecular composition of the lesion bridges bacterial growth and the recruitment of immune cells. Transcriptomic data were used to first investigate how *H. ducreyi* alters its gene expression from *in vitro* growth to the environment of a pustule (99). Comparison of biopsy samples to historic mid-log, transition, and stationary phase *in vitro* data sets showed that the *in vivo* transcriptome of *H. ducreyi* was unique (99). The genes that consistently differed between all growth stages and the biopsies are involved in essential growth pathways and suggest a shift towards anaerobiosis and the use of alternative carbon sources, such as ascorbic acid and citrate, as well as increased amino acid and metal transport, protein folding chaperones, growth arrest, and DNA replication and repair. Given that the *in vitro* and skin environments are metabolically distinct, a differential growth signature is unsurprising.

A limitation of differential gene expression studies is that only differences at the transcript level are detected and, depending on the gene, may or may not accurately reflect the other biology at the site such as post-transcriptional regulatory mechanisms or the metabolome. Since many terminally differentiated cells, such as neutrophils, generally do not contain high levels of mRNA, genes that are not well-expressed or that are post-transcriptionally regulated may not be detected in RNA-seq datasets, but their metabolic products may be detected by metabolomics. Expanding on the previous study, the *H. ducreyi* mid-log phase inoculum used to infect volunteers and biopsies of pustules along with their matched wounded skin controls were subjected to unbiased bulk RNA-seq and metabolic profiling (100). The most differentially regulated host genes in the lesion indicated a robust immune response. This metabolomics data showed that there are elevated levels of ascorbic acid as well as the presence of prostaglandins, glutamate, linoleate, and glycosphingolipids in pustules versus sham inoculated skin (100).

The matched metabolomes and transcriptomes support a richer understanding of the lesion. The elevated levels of ascorbic acid in the pustule coupled with the upregulation of *H. ducreyi* genes that metabolize ascorbic acid suggests that *H. ducreyi* is using ascorbic acid as an alternative carbon source (99, 100). Although the definitive cellular source of ascorbic acid (and other) metabolites are unknown, neutrophils take up and sequester ascorbic acid and likely release it into the abscess as they become necrotic (101). Thus, this data set helps to generate hypotheses about how different carbon sources are used by *H. ducreyi* and what host cells may be responsible for producing these carbon sources.

Since spatial information is lost during sample processing, a limitation of tissue metabolomics is that whether the metabolite is sequestered in host cells or freely available extracellularly cannot be determined. Therefore, the outstanding questions involve both sides of the host-pathogen interaction. In addition, because of the extreme difference in the amount of host material compared to the bacteria, the metabolome overwhelmingly reflects host components. For *H. ducreyi*, we hypothesize that, given the upregulation of genes to metabolize a given compound, such as ascorbic acid, coupled with the inability to synthesize the input metabolite of the pathway, *H. ducreyi* is using these metabolites as energy sources (99, 100). Generation of gene deletion mutant strains and subsequent experimental infection with these strains will confirm whether these are important carbon sources for *H. ducreyi*.

Because both the host and bacterial transcripts present in the pustule were captured from the infected samples, it was possible to use bioinformatics to generate an interaction network of host-bacterial gene clusters in an unbiased manner that were either positively or negatively correlated. Notably, genes involved in anaerobiosis were correlated with several pro-inflammatory host genes that are upregulated during infection (100). A similar strategy was used to determine an interaction network for naturally occurring lepromatous *M. leprae* biopsies; an interactome for tuberculoid *M. leprae* infection could not be established, since there were too few bacterial reads in the biopsies (4). The *M. leprae* interaction network confirmed that the type I interferon response is upregulated and that lepromatous lesions have increased bacterial metabolism (4). This was correlated to class switching in B cells (4). Application of these techniques to biopsies of lesions caused by other organisms will also elucidate whether immune system metabolism is altered along a common pathway(s) or whether infections differentially influence host metabolism. Thus, dual RNA-seq provides a tool to examine interaction networks that may identify therapeutic targets.

## Toward Uncovering Metabolic Shifts in Specific Cell Types

Understanding how the host contributes to these differences is more complicated; central carbon metabolites—namely citrate, α-ketogluterate, and succinate—have been shown to have alternative uses outside of central metabolism during inflammation (102). These metabolites can be transported out of the mitochondria to form antimicrobial metabolites, protect against oxidation, and alter protein functions through post-translational modification (102). Furthermore, whether the differences observed in *H. ducreyi* pustules are representative of changes in local cellular metabolism (e.g., from keratinocytes and fibroblasts), the infiltration of specific cell types that are high in a given metabolite, or both is not known. As such, determining whether these compounds become available to *H. ducreyi* through diffusion or secretion from live host cells or become available after host cell death and lysis is an outstanding question. Utilization of emerging spatial metabolomics approaches in the future will further advance our understanding of the lesion on the molecular level (103).

**Figure 4:**
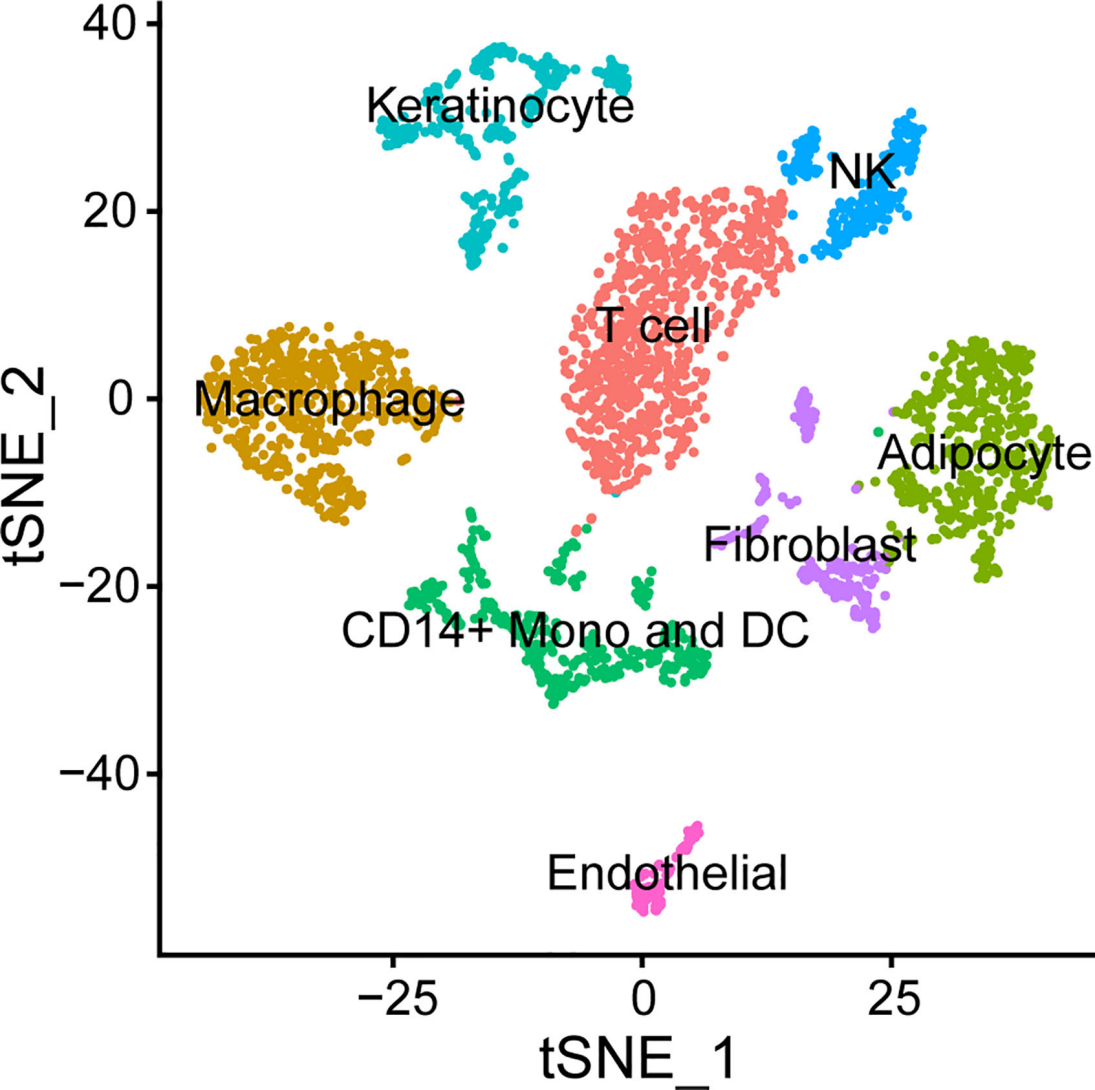
Cell types found in the *H. ducreyi* pustule by single cell RNA-seq. Cells from a pustule were dissociated and single cell sequencing was performed. Cells were clustered in Seurat (104) and identified based off the unique, abundant transcripts in each cluster.

Emerging technologies are providing new insights into specific host cell contributions to disease. Instead of restricting lesional cell characterization by using pre-selected phenotypic markers for flow cytometry, next generation sequencing provides the possibility of identifying the source of transcripts at the single cell level as well as their physical location within a lesion using spatial transcriptomics. This will allow us to better correlate the proportion of different cell types observed in previous studies (57–61); determine the cellular source(s) of differentially expressed genes observed in bulk RNA-seq studies (99, 100); and better estimate the effectiveness of the immune response in individual volunteers. In preliminary experiments, we have generated single cell suspensions and performed droplet single cell RNA sequencing on biopsies of pustules. Using Seurat (104) to cluster cells, macrophages, monocytes, DCs, T cells, NK cells, as well as endothelial cells, fibroblasts, and keratinocytes were identified in the biopsies (**Figure 4**). Interestingly, neutrophils were not detected perhaps because they are terminally differentiated and do not have high levels of mRNA. Nevertheless, the results of single cell RNA-seq studies can then be compared to our bulk transcriptomics data sets and to other publicly available single cell data sets that are available from normal and diseased human skin (2).

In previous studies, we showed that papules of pustule formers and resolvers who were re-infected for 48 hours had distinct transcription profiles that correlated with disease progression versus resolution (19). Due to the limitations of technology available at that time, we were not able to identify the cells responsible for differential transcript expression. Although we have shown that women are less susceptible to pustule formation than men, we have not been able to identify the cause(s) of the gender difference. A similar gender difference has been observed for progression from tuberculoid to lepromatous lesions for *M. leprae*, where more men progress to lepromatous lesions (90). Interestingly, some immunometabolites that are known to be regulated by sex hormones, such as prostaglandins, are upregulated in pustules (19, 100) and prostaglandin E2 has been shown to increase cytokine secretion in a monocyte-derived DC model (67). During infection, these cytokines are predicted to promote inflammatory responses in leukocytes and tissue repair in keratinocytes and fibroblasts. Single cell RNA-seq and metabolomics have the potential to unravel the differences in the tissues of pustule formers and resolvers who are re-infected and of men and women.

## The Future of Understanding Host-Pathogen Interaction in the Skin

A full understanding of how an immune response is mounted in infectious human skin diseases will require determining the metabolic environment during infection and the cell-cell signaling involved as the lesion is first formed. Because of the difficulty of obtaining sufficient numbers of cells responding to invading pathogens early in infection, the first cells to initiate the immune response is largely unknown. Thus, we do not know the *in vivo* signal(s) received by the immune system that begins the recruitment of additional leukocytes. We anticipate that new “omics” technologies will help close this knowledge gap and help the field to generate a more holistic model of skin infection.

In addition to the interaction between the pathogen and host, other environmental factors, such as the skin microbiome, may play a role in determining infection outcome. In a prospective study, the pre-infection microbiomes of sites of people who resolved experimental infection clustered together while those of pustule formers were more diverse (105). In the course of experimental infection, the microbiomes of resolvers did not change, while the microbiomes of pustule formers were driven to a similar composition; in the latter, *H. ducreyi* dominates the lesion, and the proportions of the other genera decrease substantially during disease progression (105). The pre-infection microbiomes of resolvers tend to have higher levels of *Actinobacteria*, *Firmicutes*, and *Bacteroidetes* and lower levels of *Proteobacteria* compared to pustule formers, which had higher levels of micrococci and *Staphylococcus*. Whether these microbiome differences are because the former genera actively outcompete *H. ducreyi* (e.g., secrete toxins or metabolites that *H. ducreyi* is sensitive to), are a product of environmental factors (e.g., different hygiene practices and/or products), or underlying immune responsiveness is not known. However, the data suggest that the environment of a pustule drives the microbiome to a more uniform composition.

### Future of Treatment Options for Skin Pathogens

*H. ducreyi* is currently only reliably susceptible to macrolides, quinolones, and third generation cephalosporins. For chancroid, the preferred Center for Diseases Control and Prevention regimens include a single dose of oral azithromycin, a single dose of intramuscular ceftriaxone or a 3 day course of oral ciprofloxacin (8). For CU, a single oral dose of azithromycin has been the only regimen that has been studied and is highly effective (106). Further emergence of antibiotic resistance in endemic regions may require alternative treatment strategies. The higher differential expression of genes involved in carbon source switching and hypoxia/anaerobiosis *in vivo* indicates that interfering with bacterial nutrition may be a viable treatment tactic (99, 100).

If *H. ducreyi* is found to be dependent on “unique” metabolite(s), it may be possible to develop inhibitors to enzymes used in its metabolism in *H. ducreyi*. This strategy would require that host enzymes—and potentially those of other members of the healthy skin microbiome—are not impacted by the inhibitor. If the metabolite is only used by *H. ducreyi*, then it may also be possible to prevent its uptake and essentially starve the bacteria of a carbon source. Given the emergence of (multiple) drug resistant skin pathogens such as *S. aureus* or sexually transmitted bacteria such as *N. gonorrhoeae*, limiting broad spectrum antibiotics may help to decrease the prevalence of drug resistant strains in the population. Because *H. ducreyi* infects the upper layers of the skin, topical treatments could also be developed.

Finally, experimental human infection with deletion of candidate gene targets has been used to define essential virulence genes. Identification of vaccine antigens that produce high titers of opsonizing antibody have been complicated by the low tendency of *H. ducreyi* antigens to elicit long lasting immune responses. Evaluating vaccine efficacy in animal models is challenging because animal models lead to pathogen clearance and/or require very high dosages for infection. Nevertheless, two vaccine candidates have been studied in the porcine ear model. They are Class I specific and are most likely ineffective against Class II strains because the evaluated genes are among the genes with the most sequence diversity between Class I and Class II strains (107, 108). Therefore, these results are not wholly unanticipated, but suggest that targeting essential specific outer membrane proteins may not be as fruitful of a strategy as originally thought.

## Conclusions

The ability to infect humans with *H. ducreyi* has been instrumental in exploring host-pathogen interaction at the tissue level. Nearly thirty years of human challenge experiments are helping to confirm previous studies as well as guide us toward new biological insights for future study on the cellular and molecular levels. This model is beneficial for both the skin immunology community, since it is one of the few human experimental infection models that captures the cells and metabolites of the lesion microenvironment. Thus, a rich understanding of cell types in the lesion, immune activation, and lesion architecture is achieved.

Advancements in “omics” technologies afford researchers a more holistic view of host-pathogen adaptions during infection. Bacterial virulence has expanded from primarily being thought of as the result of extracellular and secreted bacterial effectors to including how metabolism—both in the host and the bacteria—influence infection outcomes. Increased awareness of differences in microbiomes (105) and nutrient availability *in vivo* (99, 100) has highlighted that bacteria differentially regulate genes in the context of the host response and alterations in nutrient availability.

As we investigate skin inflammatory responses, we must also acknowledge that responses can differ not only by pathogen but also potentially by skin site. For other inflammatory conditions such as psoriasis, single cell RNA-seq revealed that while most dysregulated genes in psoriasis are independent of location on the body, some differentially expressed genes are unique to where psoriatic lesions are located (2). Most notably, different T cell subsets are activated based on location, which may influence therapeutic intervention strategies and outcomes (2). Similarly, location of disease may be of particular interest in *H. ducreyi* infection, since it infects both cutaneous lower leg and arm skin as well as genital skin. How or if the immune response significantly differs in these two locations has not been studied; however, the relationships between *H. ducreyi* and host cells are similar in experimental and natural infection (1). The use of clinical samples in infectious disease will be instrumental in elucidating the mechanisms behind various skin pathologies and in appreciating both inter-person and intra-person response diversity. As more data become available for different diseases, we anticipate that common immune response pathways will emerge, giving us a better understanding of how skin infections and the immune system interact.

## Author Contributions

JB, BG, and SS conceptualized the manuscript. JB and BG wrote the original draft. LC analyzed transcriptomic data. JB, BG, LC, and SS revised the final draft of the manuscript. All authors contributed to the article and approved the submitted version.

## Funding

JB and BG were supported by T32AI007637. This work was supported by grant UL RR052761 from the Indiana Clinical and Translational Sciences Institute and the Indiana Clinical Research Center and by R01AI134727 and R01AI137116 from the National Institute of Allergy and Infectious Diseases to SS.

## Conflict of Interest

The authors declare that the research was conducted in the absence of any commercial or financial relationships that could be construed as a potential conflict of interest.
